# Examining the validity and reliability of the Arabic translated version of the depression and somatic symptoms scale (A-DSSS) among the Lebanese adults

**DOI:** 10.1038/s41598-024-55813-z

**Published:** 2024-03-05

**Authors:** Ali Ismail, Alfred Chabbouh, Elie Charro, Jad El Masri, Maya Ghazi, Najwane Said Sadier, Linda Abou-Abbas

**Affiliations:** 1 Faculty of Medical Sciences, Lebanese University, Beirut, Lebanon; 2 Faculty of Medical Sciences, Neuroscience Research Centre, Lebanese University, Beirut, Lebanon; 3 Department of Anatomy, Cell Biology and Physiological Sciences, American University of Beirut, Beirut, Lebanon; 4 College of Health Sciences, Abu Dhabi University, Abu Dhabi, UAE; 5 INSPECT-LB (Institut National de Santé Publique Epidémiologie Clinique et Toxicologie-Liban), Beirut, Lebanon

**Keywords:** Depression, Somatic symptoms, Validity, Reliability, Arabic translation, Lebanese adults, Psychology, Medical research

## Abstract

The prevalence of depression is high worldwide, and somatic symptoms are known to be one of the most debilitating aspects of depression. However, clinicians often face challenges in accurately assessing this comorbidity. To address this issue, the Depression and Somatic Symptoms Scale (DSSS) was developed as a self-administered scale that can diagnose both depression and somatic symptoms. The objective of this study is to evaluate the validity and reliability of the Arabic-translated version of the DSSS (A-DSSS) in a sample of Lebanese adults, as well as to explore its associated factors. A cross-sectional study was conducted over a period of one month, from February to March 2023, and involved a sample of 422 participants who were aged 18 years or older. Participants completed a questionnaire that included various measures, including demographic characteristics, alcohol and smoking habits, physical activity history, as well as two scales: the Patient Health Questionnaire-9 (PHQ9) scale and the A-DSSS scale. The A-DSSS showed high internal consistency (Cronbach’s alpha = 0.936), strong test–retest reliability (ICC of 0.988 with CI 0.976–0.994; p < 0.001), and a three-factor structure consistent with previous research. Convergent validity was supported by a significant correlation with the PHQ-9. Stepwise linear regression revealed that engaging in physical activity and increasing calorie consumption (as measured by MET-min/week score) were associated with a significant decrease in the A-DSSS total score and subscales. However, a significant increase in the A-DSSS total score was seen in the female gender in comparison for male gender. The A-DSSS revealed good psychometric properties and may be a useful tool for assessing depression and somatic symptoms in this population. The study also identified potential factors associated with depression and somatic symptoms, such as physical activity, calorie consumption, and gender, which may have implications in addressing depression and somatic symptoms for future interventions and clinical practice.

## Introduction

Depression is a very common condition worldwide, affecting around 3.8% of the population and 5.0% of adults, with approximately 280 million individuals’ worldwide experiencing depression^1^. One of the most disabling manifestations of depression is the presence of somatic symptoms, which although not fully understood in terms of pathophysiology, and that lead to significant distress^1^. Somatic symptoms are frequent complaints in people with depression and they can be the primary reason for seeking medical care, especially in primary healthcare settings^2^. Studies have shown that two-thirds of individuals with depression experience somatization^3^ and that 69% of depressed patients in primary care manifest somatic symptoms as their main reason for seeking medical advice^4^.

Persistent somatic symptoms can complicate the diagnosis and treatment of depression, sometimes leading to confusion or masking of the condition and misdiagnosis^5^. Additionally, these symptoms may increase the risk of relapse, as they can persist even after initial treatment and contribute to ongoing distress^6–8^. Individuals with major depressive disorder and somatization tend to experience higher levels of depressive symptoms and longer, more frequent depressive episodes compared to those without somatization^9^. Thus, somatic symptoms have a significant impact on the diagnosis, treatment, and prognosis of depression and therefore require accurate assessment tools leading to effective management and treatment strategies^6^.

Many existing depression measurement scales was developed to assess somatic symptoms. Traditional scales, such as the Hamilton Depression Rating Scale (HAMD)^10^, consolidate diverse physical symptoms into just a few questions. The Patient Health Questionnaire-15 (PHQ-15)^11^ attempts to address this by incorporating 15 items to evaluate somatic symptoms and their impact on the individual. The Somatic Symptom Scale-8 (SSS-8)^12^, a condensed version of the PHQ-15 with items related to menstrual problems, sexual problems, and fainting removed, assesses eight distinct physical symptoms to gauge the distress caused by common physical symptoms. However, a common issue with these scales is the insufficient number of questions, and both the PHQ-15 and SSS-8 focus solely on the degree of somatization, lacking a comprehensive tool for accurately assessing and monitoring individual physical symptoms.

Given the importance of exploring somatic symptoms in depression, Hung et al. created the Depression Somatic Symptoms Scale (DSSS)^13^, a self-reported depression scale with 22 items and two subscales: 12 items for the depression subscale (DS) and 10 items for the somatic subscale (SS). The latter includes 5 items specifically related to pain. This scale was first developed in Chinese population and has been translated and validated on a sample of Taiwanese outpatients with Major Depressive Disorder (MDD)^13^.

In the Arabic-speaking population, no study has yet examined the psychometric properties of an Arabic version of the DSSS, the majority of tools that are used they lack the presence of somatic symptoms assessment, and the need of a tool that contain both aspect, the depression and somatic symptoms, its important in the future research projects in the Arabic country. Therefore, the aim of this study is to examine the validity and reliability, including internal consistency, convergent and structural validity of the Arabic translated version of the DSSS (A-DSSS) in a sample of Lebanese adults.

## Methods

### Translation process

After obtaining permission from the corresponding author to translate the original version of DSSS into the Arabic language, we performed the translation of the DSSS scale according to the guidelines proposed by Beaton et al.^14^. Firstly, two independent professional translators forward translated the DSSS from English to the Arabic. Secondly, the two produced drafts were compared and until a final translation was agreed upon through a consensus process. Thirdly, two different independent translators who were blinded to the original English version of DSSS, back-translated the Arabic version. The two back-translated versions were examined by a professional committee which deemed the translations faithful to the original English version. Finally, the content validity of the final draft version was assessed by a committee of experts consisting of a psychiatrist and three licensed psychologists who were not involved in the initial translation. This committee was asked to rate the relevance of each item on a 4-point Likert scale from 1 as ‘‘not relevant’’ to 4 as ‘‘very relevant’’. Content validity for each item (CVI) was calculated as the proportion of experts rating either three or four (quite relevant and very relevant, respectively), divided by the total number of experts. All items with a CVI rating of 0.8 or above were retained. As such, no items were discarded from our scale. A pre-final version of the translation was drafted and administered as a pilot study to 15 random individuals to assess the clarity and understanding of the items. No problems were reported, and therefore no modifications were made to the scale.

### Study design and participants

A cross-sectional study was carried out over a period of one month, extending from april to May 2023. All the participants were recruited through convenience sampling from all Lebanese governorates. Persons aged above 18 and those who could read Arabic were included in our study.

The guidelines suggest that a sample size of 5–10 participants per scale item is sufficient for establishing the validity and reliability of a scale^15^. Since the A-DSSS has 22 items, we needed at least 110 to 220 participants. Responses were collected from 422 adults.

### Data collection

The questionnaire used in the study was in Arabic and consisted of four sections. The first section included questions about socio-demographics characteristics (age, gender, governorate, educational level, and marital status), the second section included questions about health behavior including: 1-alcohol consumption, 2- smoking status and history (current, past, non-smoker), and 3- physical activity status. The third section consisted of the Patient Health Questionnaire-9 (PHQ-9) Arabic version, and the last section comprised the translated Arabic version of the Depression and Somatic Symptoms Scale (A-DSSS).

### Study measurements

1. Alcohol consumption The Alcohol Use Disorder Identification Test (AUDIT-C), a 3-item instrument that can help identify patients who are drink hazardously or have active alcohol use disorders^16^.
2. Smoking history assessed by pack-years calculated by multiplying the number of cigarettes smoked per day by the number of smoking years and dividing by 20^17^.
3. Physical activity status was assessed using the metabolic equivalent (MET)-minute score, which is calculated by adding the duration of physical activity in minutes to the frequency of activity in days. MET is the ratio of energy consumption during activity to energy consumption at rest^18,19^.

To calculate the MET-min/week score, we used the following formulas:

- Walking MET-min/week = 3.3 × walking minutes × number of walking days
- Moderate MET-min/week = 4.0 × moderate activity minutes × number of days with moderate severity activities
- Severe MET-min/week = 8.0 × severe activity minutes × number of days with severe activities

The total MET-min/week score was calculated by adding the walking, moderate, and severe MET-min/week scores. Based on the total MET score, participants were classified into three activity levels:

- Low level: ≤ 600 MET-min/week.
- Moderate level: 600–3000 MET-min/week.
- High level: ≥ 3000 MET-min/week.

4. The PHQ-9 is a self-report scale used to assess depressive symptoms. It contains 9 questions that cover the 9 diagnostic criteria for major depressive disorder mentioned in the DSM-V^20^. The scale assesses the participants' mood within the last two weeks. Each item is graded from 0 (absence of symptom) to 3 (presence of symptom nearly every day) giving a total score ranging from 0 to 27.
5. The DSSS is a self-reported scale consisting of 22 items that evaluate depressive and somatic symptoms. It includes a depression subscale (DS) with 12 items, including three vegetative symptoms and fatigue, which are not included in SS because they are criteria for major depressive episodes and are also included in the HAMD scale. Somatic subscale (SS) with 10 items, five of which are part of the pain subscale (PS). The researchers chose the somatic items that could indicate the severity of depression, predict the onset of depression, or have a significant impact on clinical practice or health-related quality of life. They also selected symptoms that were commonly reported in previous studies of depression, as well as those that improved the reliability of the Depression Somatic Symptom Scale (DSSS) measure. Each item is scored on a Likert scale ranging from 0 to 3 (absent, mild, moderate, and severe), with a maximum score of 36^13^.

### Ethical consideration

This study was conducted in accordance with the Declaration of Helsinki, the Institutional Review Board of Al Sahel Hospital approved the study with a reference number (5/2023). Participants were provided with a clear explanation of the purpose and procedures of the study before giving their consent to participate. Informed consent was obtained from all participants, only participants who read, understood, and consented to filling the online questionnaire were allowed to participate in the study. To ensure participants' anonymity and confidentiality of information, no personal identifying information was collected during the study. Participants were informed that their participation was voluntary, and they had the right to withdraw at any time without consequences.

### Statistical analysis

Data analysis was conducted using SPSS version 22.0. Descriptive statistics were used to report means and standard deviations (SD) for continuous variables, and frequencies with percentages for categorical variables. Baseline characteristics and health behaviors, were compared between males and females using chi-square for categorical variables and student t-test for continuous variables.

The internal consistency of the A-DSSS was assessed using Cronbach’s alpha coefficient. A coefficient above 0.7 was considered indicative of good internal consistency^21^. Test–retest reliability was assessed through the interclass correlation coefficient (ICC; average measure) between the total score of A-DSSS in a subsample of 30 participants who filled the form twice, initially and subsequently after two weeks. Good reliability was noted when ICC > 0.7^22^.

To assess convergent validity, Pearson correlation was used to examine the relationship between the DSSS total score, sub-scales scores, and the total score of PHQ-9. For construct validity, we randomly divided the sample into two groups. Exploratory factor analysis (EFA) using principal components analysis (PCA) with Varimax rotation was conducted on the first half sample (n = 211) to explore the A-DSSS factor structure. Sampling adequacy was assessed by the Kaiser–Meyer–Olkin (KMO) measure and Bartlett’s test of sphericity. The number of factors retained in the scale was determined based on Eigenvalues greater than 1,and factors are retained if they showed communalities above the cut-off scores of 0.25 and 0.4^23^.

Confirmatory factor analysis (CFA) was conducted using the AMOS software version 22 on the second half of the sample. The goodness-of-fit of the models was assessed using Chi-square (χ^2^) and degrees of freedom (df), Root Mean Square Error of Approximation (RMSEA), and Comparative Fit Index (CFI).

Independent t-tests and ANOVA were used to assess the correlation between the A-DSSS total score and sub-scale scores with socio-demographic characteristics, smoking, alcohol consumption, physical activity levels, lifestyle, and healthy behaviors. The adjusted odds ratios (ORs) with 95% confidence intervals (CIs) were calculated. The goodness-of-fit of the model was assessed using the Hosmer–Lemeshow test. All statistical tests were two-sided, and the significance level was set at 0.05.

## Results

### Baseline characteristics of the study participants

Table 1 represents the baseline characteristics of the study participants. A total of 422 participants were enrolled in this study, with a majority of women (67.3%). The average age of the sample was 25.08 with a standard deviation of 8.4. The majority of participants were university students (79.2%), single (86.5%), and more than half were unemployed (51.4%). Most participants were non-smokers (85.1%), with an average pack per year of 6.55, and non-alcohol drinkers (70.9%), with an average AUDIT-C score of 1.97.

**Table 1:** Baseline characteristics of the study participants.

Characteristics	All (N = 422)
Age (years) mean (SD)	25.08 (8.43)
Gender n (%)	
Male	138 (32.7)
Female	284 (67.3)
Educational level n (%)	
Primary or no education	5 (1.2)
Secondary	83 (19.6)
University	334 (79.2)
Marital status n (%)	
Married	57 (13.5)
Single	365 (86.5)
Employment status n (%)	
Full time	105 (24.9)
Part time	100 (23.7)
Unemployed	217 (51.4)
Smoking status n (%)	
Non smoker	359 (85.1)
Past smoker	16 (3.8)
Current smoker	47 (11.1)
Pack per year mean (SD)	6.55 (12.13)
Alcohol intake n (%)	
Yes	123 (29.1)
No	299 (70.9)
Audit-C mean (SD)	1.97 (1.63)
Physical activity n (%)	
No	217 (51.42)
Yes	205 (48.57)
Low physical activity (< 600 MET-min/week)	42 (20.48)
Moderate physical activity (600–3000 MET-min/week)	129 (62.92)
High physical activity (> 3000 MET-min/week)	34 (15.58)

*n* frequency, *SD* Standard deviation, *MET* metabolic equivalent, *min* minute.

Around 44% of the total participants reported regular exercise in their life with a mean of physical activity of (1790and SD = 149.11 MET-min/week). Of those who exercise regularly, the majority (62.9%) reported moderate physical activity, with an average of 1650.55 and SD = 677.09 MET-min/week. Less than 21% reported low physical activity, with an average of 441.14 and SD = 120.3 MET-min/week, while approximately 16.5% reported high physical activity with an average of 3990.52 and SD = 694.43 MET-min/week.

Results of the assessment of the used PHQ9 and A-DSSS scales (and its sub-scales) are shown in Table 2. The mean A-DSSS score was 22.62 (± 13.84) in the total sample.

**Table 2:** Clinical characteristics of the study participants.

	Mean (SD)
A-DSSS total score	22.62 (13.84)
A-DSSS depression subscale score	13.7 (8.29)
A-DSSS somatic subscale score	8.88 (6.59)
A-DSSS pain subscale score	4.84 (3.57)

*A-DSSS* Depression and Somatic Symptoms scale Arabic version, *SD* standard deviation.

### Psychometric properties of the A-DSSS

#### Reliability of the A-DSSS

The internal consistency of the A-DSSS was assessed using Cronbach’s alpha coefficients and composite reliability (CR) coefficients. The results showed high internal consistency, with a Cronbach’s alpha of 0.936. The corrected–item to total correlation coefficients varied from 0.41 to 0.749. Removal of any item from the construct did not significantly affect Cronbach’s alpha, which ranged between 0.931 and 0.938 (Table 3). The test–retest reliability was also assessed, and the interclass coefficient showed a strong reproducibility, with an ICC of 0.988 (CI 0.976–0.994; p < 0.001), indicating good reliability.

**Table 3:** Internal consistency of the A-DSSS.

	Scale mean if item deleted	Scale variance if item deleted	Corrected item-total correlation	Cronbach's alpha if item deleted
A-DSSS1: headache	21.5758	178.193	0.529	0.935
A-DSSS2: loss of interest in daily or leisure activities	21.3389	174.320	0.637	0.933
A-DSSS3: tightness in the chest	21.8081	176.355	0.618	0.934
A-DSSS4: insomnia	21.6445	176.054	0.536	0.935
A-DSSS5: muscle tension	21.5142	174.255	0.607	0.934
A-DSSS6: irritable mood	21.1232	174.811	0.632	0.933
A-DSSS7: back pain	21.4479	176.509	0.522	0.935
A-DSSS8: unable to feel happy or decreased ability to feel happy	21.3057	173.182	0.677	0.933
A-DSSS9: dizziness	21.9763	176.755	0.605	0.934
A-DSSS10: depressed mood or tearful	21.4408	171.687	0.738	0.932
A-DSSS11: chest pain	22.0664	176.385	0.668	0.933
A-DSSS12: feelings of self-reproach or guilt	21.4455	172.452	0.629	0.933
A-DSSS13: neck or shoulder pain	21.3436	175.010	0.544	0.935
A-DSSS14: loss of interest in sex	21.9052	179.616	0.411	0.937
A-DSSS15: shortness of breath or difficulty breathing	21.9882	176.620	0.633	0.933
A-DSSS16: anxious or nervous	21.1066	171.530	0.749	0.931
A-DSSS17: soreness in more than half of the body’s muscles	21.8531	174.624	0.645	0.933
a-dsss18: unable to concentrate	21.2536	170.674	0.747	0.931
A-DSSS19: palpitations or increased heart rate	21.8009	174.597	0.644	0.933
A-DSSS20: thoughts of death or suicidal ideas	22.2180	178.845	0.545	0.935
A-DSSS21: fatigue or loss of energy	21.0616	172.319	0.704	0.932
A-DSSS22: decreased appetite or loss of appetite	21.9194	179.224	0.493	0.935

*A-DSSS* Depression and Somatic Symptoms scale Arabic version.

#### Construct validity of the A-DSSS

The exploratory factor analysis of the DFS-A scale showed a KMO measure of 0.936 which was above the commonly accepted value of 0.60^23^ as well as a highly significant Bartlett’s Test of sphericity (χ^2^ = 2720.798, df = 231, p-value < 0.0001) indicating that factor analysis was suitable (Table 4).

**Table 4:** Exploratory factorial analysis of the A-DSSS.

	Component			h2 communalities
	1	2	3	
A-DSSS 2: loss of interest in daily or leisure activities	0.811			0.713
A-DSSS 4: insomnia	0.431			0.374
A-DSSS 6: irritable mood	0.721			0.608
A-DSSS 8: unable to feel happy or decreased ability to feel happy	0.784			0.695
A-DSSS 10: depressed mood or tearful	0.77			0.717
A-DSSS 12: feelings of self-reproach or guilt	0.716			0.597
A-DSSS 14: loss of interest in sex	0.716			0.258
A-DSSS 16: anxious or nervous	0.715			0.708
A-DSSS 18: unable to concentrate	0.683			0.699
A-DSSS 20: thoughts of death or suicidal ideas	0.519			0.409
A-DSSS 21: fatigue or loss of energy	0.748			0.655
A-DSSS 22: decreased appetite or loss of appetite	0.432			0.357
A-DSSS 3: tightness in the chest		0.689		0.606
A-DSSS 9: dizziness		0.484		0.489
A-DSSS 11: chest pain		0.778		0.734
A-DSSS 15: shortness of breath or difficulty breathing		0.835		0.787
A-DSSS 19: palpitations or increased heart rate		0.677		0.614
A-DSSS 1: headache			0.531	0.435
A-DSSS 5: muscle tension			0.729	0.668
A-DSSS 7: back pain			0.762	0.641
A-DSSS 13: neck or shoulder pain			0.805	0.688
A-DSSS 17: soreness in more than half of the body’s muscles			0.753	0.697
Eigenvalue	10.025	1.813	1.308	
Percentage of explained variance	45.57%	8.243%	5.947%	

*A-DSSS* Depression and Somatic Symptoms scale Arabic version.

Three factors had eigenvalues above 1.0, The scree plot of the Eigenvalues revealed a three-factor structure of the DFS-A scale: the first factor was related to depressive symptoms which included all the 12 items of the original 12-item depression sub-scale^2,4,6,8,10,12,14,16,18,20–22^ and accounted for 45.57% of the scale’s total variance; the second factor represented the somatic symptoms of depression and included items^2,9,11,15,18^ and accounted for 8.243% of the scale’s total variance; and the third factor contained items related to pain symptoms and accounted for 5.947% of the scale’s total variance. All factors were retained because they showed communalities above the cut-off scores of 0.25 and 0.4^23^. Factor loadings for each item are presented in Table 3. However, random eigenvalues derived from parallel bootstrapping showed that two factors would have been selected (eigenvalues of 1.62 and 1.76).

**Figure 1:**
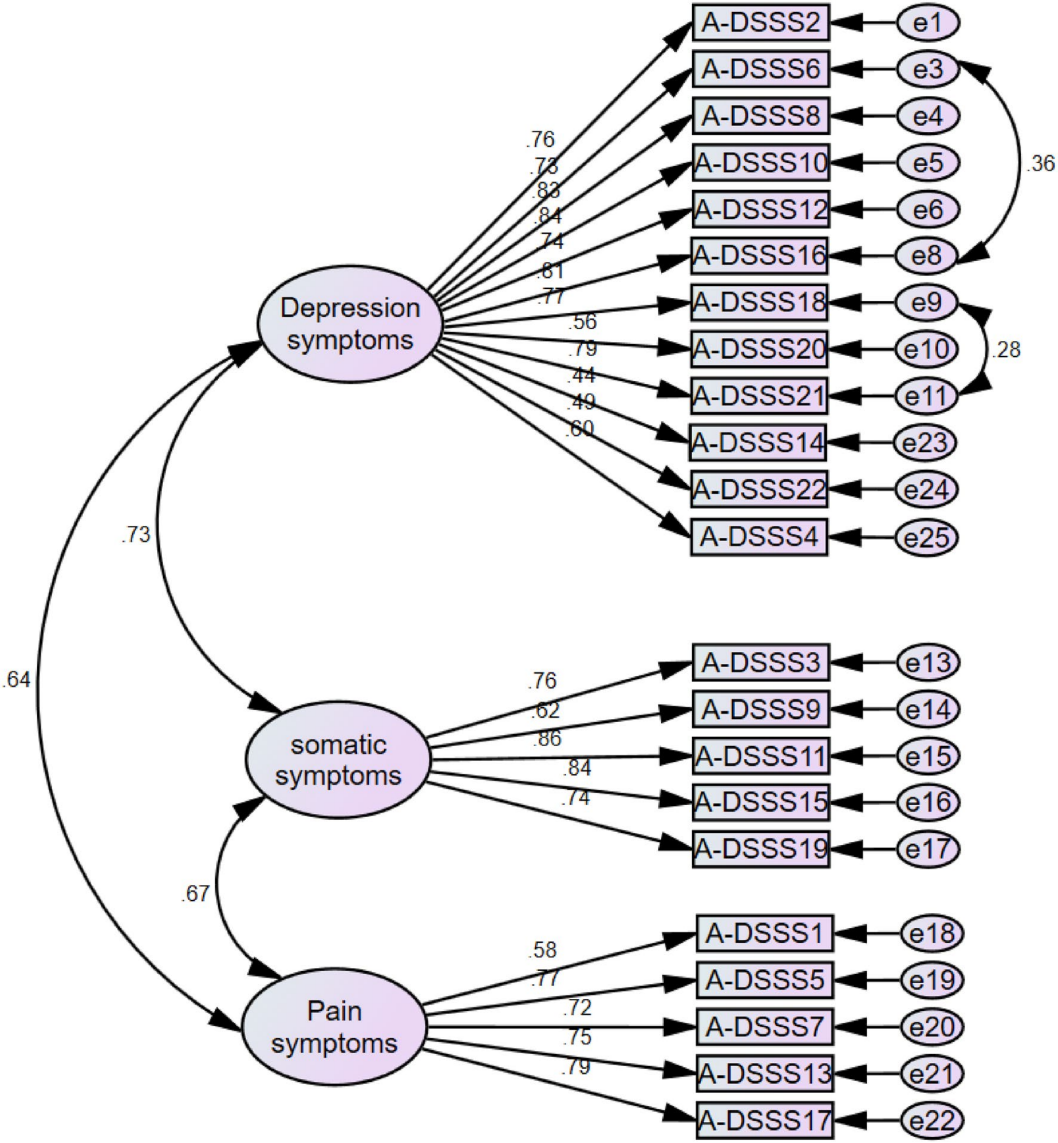
Three-factor model of the Arabic version of A-DSSS, A-DSSS1 till A-DSSS22 are observed variables; err 1 till err 22 are unobserved variables.

Finally, the A-DSSS multidimensionality model was determined via a confirmatory factor analysis showed in Fig. 1. As the EFA suggested a 3-factor solution, the inspection of this model fit well with the data and showed a satisfactory fitness. The goodness of fit statistics was NFI = 0.854, CFI = 0.919 above the cut-off value 0.9, RMSEA = 0.071 and χ^2^/df = 1.94 below the cut-off values RMSEA < 0.08, and χ^2^/df ≤ 2.0^24^. After adding paths from A-DSSS 6 and 16, A-DSSS 18 and 21 caused an improvement of the fit indices. The goodness of fit statistics became NFI = 0.863, CFI = 0.928, and RMSEA = 0.06.

#### Convergent validity of the A-DSSS

Spearman correlation between the PHQ-9 score and the A-DSSS total score and its subscales scores show a statistically significant correlation with a coefficient ranging from 0.297 to 0.457 (p < 0.001) as detailed in Table 5, indicating adequate convergent validity of the A-DSSS.

**Table 5:** Spearman correlation between PHQ9 and DSSS scores.

Measure	Spearman coefficient	P value
A-DSSS total score	0.457	< 0.001
A-DSSS depression subscale score	0.464	< 0.001
A-DSSS somatic subscale score	0.363	< 0.001
A-DSSS pain subscale score	0.297	< 0.001

*A-DSSS* Depression and Somatic Symptoms scale Arabic version.

### Careless analysis

In order to ensure the reliability of participant responses, we meticulously examined the possibility of careless responding. Following the methodology suggested by Meade and Craig^25^, and Curran et al.^26^, we incorporated an infrequency scale within the survey items to identify respondents who might have provided responses randomly or without due consideration. To assess the robustness of our dataset, we conducted a thorough post hoc analysis using a multi-faceted approach proposed by Ward and Meade^27^. While the ideal prior screening step before data collection was not feasible in our case, we employed various measures to identify potential issues in participant responses. Post hoc analysis on the collected data was done by Utilizing longstring as an effective tool for identifying invariant responses and Mahalanobis distance, along with even–odd consistency (personal reliability), as optimal measures for discerning random responses within our collected data was pivotal. Long-string computation served as an effective tool for detecting invariant responses, especially extreme straight lining, where respondents consistently selected the same option. Establishing a definitive cut-off value for long-string analysis presented challenges due to the absence of a specific threshold for excessively prolonged identical responses. Nevertheless, our dataset analysis revealed meaningful insights, with only 17 responses exceeding the cut-off range of 6 to 14 out of a mean of 4.93, a standard deviation (SD) of 3.75, a minimum of 12, and a maximum of 22. Additionally, the even–odd index methodology involved segregating scale items into two subscales based on even and odd numbering. Both subscales exhibited highly similar Cronbach's alpha values, indicating internal reliability consistency. The substantial correlation of 0.774 between these subscales, supported by a robust Spearman Brown coefficient of 0.873, indicated meticulous completion of the questionnaire, surpassing the 0.30 threshold proposed by Jackson^28^. Furthermore, the Mahalanobis distance, proposed by Mahalanobis in 1936^29^, was employed as a multivariate outlier statistic considering the correlational structure between items. The calculated mean of 199, standard deviation (SD) of 3.97, minimum of 0.00043, and maximum of 42.54 provided insights into the distribution of responses. The p-value analysis revealed that only 7 responses exhibited a p-value less than 0.001, identifying these participants as outliers in our dataset. This comprehensive approach ensures the robustness and reliability of our data, contributing to the validity of our study outcomes.

### Factors associated with A-DSS and its subscales

The results of the bivariate analysis are summarized in Table 6. Females scored significantly higher on the A-DSSS and its sub-scales (p < 0.01). Only physical activity was negatively correlated with A-DSSS, DS, SS, and PS scores. The mean PS score was also higher in current smokers (6.02 vs 4.63, p < 0.05). A stepwise linear regression, using the A-DSSS total score as a continuous variable summarized in Table 7, showed that doing physical activity, and an increase in calorie consumption (MET-min/week score) decrease significantly the DSSS total score (Beta = − 5.657, and Beta = − 0.002 respectively). On the other hand, being a female would significantly increase A-DSSS total score (Beta = 3.751, P < 0.001).

**Table 6:** Bivariate analysis of the A-DSSS total scale and its subscales with demographic and lifestyle behaviors (N = 422).

Characteristics	A-DSSS		DS		SS		PS	
	Mean (SD)	p-value	Mean (SD)	p-value	Mean (SD)	p-value	Mean (SD)	p-value
Gender								
Male	20.10 (13.77)	0.009*	12.35 (8.46)	0.016*	7.74 (6.34)	0.013*	4.15 (3.49)	0.006*
Female	23.85 (13.73)		14.41 (8.13)		9.43 (6.64)		5.17 (3.59)	
Educational level								
Primary/no education	32.8 (11.09)	0.119	19.4 (8.44)	0.181	13.4 (3.2)	0.132	7.8 (2.28)	0.157
Secondary	24.1 (15.47)		14.51 (9.11)		9.66 (7.23)		4.97 (3.64)	
University	22.08 (13.39)		8.05 (0.44)		8.61 (6.43)		4.76 (3.55)	
Marital status								
Single	22.79 (13.77)	0.52	14.03 (8.29)	0.065	8.75 (6.53)	0.334	4.75 (3.55)	0.202
Married	21.52 (14.34)		11.85 (8.09)		9.66 (6.96)		5.4 (3.66)	
Employment status								
Full time	22.77 (13.71)	0.991	13.32 (7.69)	0.227	9.44 (7.02)	0.643	5.23 (3.75)	1.223
Part time	22.64 (14.19)		13.67 (8.46)		8.97 (6.88)		4.96 (3.81)	
Unemployed	22.54 (13.8)		13.98 (8.51)		8.56 (6.23)		4.59 (3.35)	
Smoking								
Non-smoker	22.34 (13.6)	0.605	13.77 (8.25)	0.971	8.56 (6.39)	0.06	4.63 (3.44)	0.016**
Past smoker	24.43 (12.9)		13.75 (6.85)		10.68 (7.04)		6.06 (4.09)	
Current smoker	24.17 (15.45)		13.46 (9.12)		10.7 (7.58)		6.02 (4.07)	
Alcohol intake								
Yes	22.64 (13.86)	0.987	13.31 (7.99)	0.498	9.32 (6.88)	0.376	5.2 (3.87)	0.182
No	22.61 (13.86)		13.91 (8.41)		8.69 (6.47)		4.69 (3.43)	
Physical activity								
Yes	19.75 (13.02)	< 0.001*	11.58 (8.27)	< 0.001*	8.16 (6.29)	0.03*	4.35 (3.41)	0.006*
No	25.34 (14.07)		15.78 (7.76)		9.55 (6.80)		5.3 (3.66)	

*p-value < 0.05 is considered significant.

*N* Frequency, **post hoc test demonstrates a significant p value between current smoker and non-smoker.

*A-DSSS* The Arabic version of the depression and somatic symptoms scale, *DS* depression subscale, *SS* somatic subscale, *PS* pain subscale, *PHQ9* the Arabic version of the Public Health Questionnaire.

**Table 7:** Linear regression taking the A-DSSS total score and subscales scores as dependent variable.

	Unstandardized Beta	Standardized Beta	p-value	95% CI
A-DSSS total score				
Female gender	3.751	0.127	0.009*	0.946; 6.555
Physical activity (yes)	− 5.657	− 0.204	** < 0.001**	− 8.273; − 3.040
MET-min/week	− 0.002	− 0.171	** < 0.001**	− 0.003; − 0.001
Current smoker	1.738	0.040	0.418	− 2.475; 5.951
DS total score				
Female gender	1.507	0.085	0.088	− 0.225; 3.239
Physical activity (yes)	− 3.845	− 0.232	0.001*	− 6.060; − 1.629
MET-min/week	− 0.002	− 0.171	** < 0.001**	− 0.003; − 0.001
Current smoker	0.986	0.037	0.441	12.881; 16.304
SS total score				
Female gender	1.849	0.132	0.01*	0.449; 3.248
Physical activity (Yes)	− 1.131	− 0.086	0.215	− 2.921; 0.659
MET-min/week	− 0.001	− 0.202	** < 0.001**	− 0.002; − 0.001
Current smoker	2.929	0.140	0.005*	6.588; 9.353

Significant values are in bold.

*Significant p value < 0.05.

Variables entered in the linear regression: age, gender, marital status, education level, physical activity, AUDIT-C score, Pack per year score.

*A-DSSS* The Arabic version of the depression and somatic symptoms scale, *DS* depression subscale, *SS* somatic subscale.

## Discussion

Somatic symptoms of depression are often underestimated and not fully considered in the assessment and management of depression, despite their significant impact on various aspects of the condition. These symptoms, which include physical manifestations such as pain, fatigue, and changes in appetite or sleep, can have a profound effect on an individual's overall well-being and functioning. However, they are sometimes overlooked or downplayed in clinical practice, leading to incomplete understanding and management of depression^13^. In this study, we aimed to translate the A-DSSS and evaluate its psychometric properties in a sample of Lebanese adults. In addition, we sought to evaluate the factors associated with the A-DSSS and its subscales. The study found that the Arabic A-DSSS scale had adequate validity and reliability in the sample of Lebanese adults and could be used as a reference in future studies and clinical practices. The results also suggest potential associations between gender, physical activity, smoking status, and somatic symptoms of depression, which may be a step stone for future research studies and strategies involved in understanding and managing depression in the Lebanese populations.

The Arabic DSSS demonstrated good internal consistency with a Cronbach's alpha of 0.93. This indicates that the items in the scale were consistently measuring the same underlying construct, and the scale was reliable in assessing depression and somatic symptoms in the studied population. The Cronbach's alpha value of 0.93 for the A-DSSS is similar to previous studies that have assessed the original DSSS developed in Taiwan (Cronbach's alpha of 0.87)^12^ and the English version developed in the US (Cronbach’s alpha of 0.91)^2^. This consistency in reliability across different language versions of the scale suggests that the Arabic DSSS is comparable to the original and translated versions in terms of internal consistency.

The findings of the study revealed that the Arabic version of the DSSS showed a three-dimensional model, which differed from the original Taiwanese scale and its English translation, which demonstrated a two-dimensional model^2,13^. The three-dimensional model, which explained about 60% of the variance, was considered the optimal model based on confirmatory factor analysis (CFA) in this study. The three-dimensional model appeared to differentiate somatic symptoms (SS) from pain symptoms (PS) in the Arabic DSSS, which was unlike the original DSSS validated in Taiwan^13^. It is worth noting that the validated American version of the DSSS did find a three-factor solution but opted for the two-model solution as a better fit^2^, while another Taiwanese study argued for a unidimensional model of the DSSS^30^. This suggests that there may be cultural and contextual differences in how depression and somatic symptoms are expressed and experienced in Arabic-speaking populations compared to the original Taiwanese and English-speaking populations. Cultural beliefs around mental health and societal structures could impact the manifestations of depressive, somatic, and pain symptoms. For instance, there is evidence that individuals in non-Western societies, including Arabs, tend to report "medically-unexplained symptoms" at higher rates^31–33^. This suggests that the expressions of somatic and pain complaints may be influenced by cultural factors such as language, expression, religion, and stigma.

Stigma, in particular, can play an important role in the expression of psychological symptoms, as one study on depression in Arabic-speaking refugees revealed that the expression of psychological symptoms of depression, but not somatic ones, were associated with high levels of stigma^34^. This highlights the importance of socio-cultural contexts in shaping the expression of psychological symptoms. Therefore, future research should further explore the sociocultural determinants of depressive symptoms in different populations, including Arabic-speaking populations. It is also recommended that future research conduct separate factor analysis, as the structure of the DSSS may vary depending on the population studied. Even within different Arabic-speaking countries, adjustments may be required for the scale to fit certain sociocultural specificities. This underscores the need for cultural sensitivity in the assessment of depressive and somatic symptoms, and the importance of considering cultural factors when interpreting research findings related to symptom measures.

In terms of convergent validity, the Arabic DSSS was highly and positively correlated with PHQ-9 scores, the highest correlation being with the depression subscale. This is expected as both scales are developed to assess for depression yet differ in structure and which symptoms domains they assess. DSSS, as conceptualized, seems to provide a better assessment of somatic and pain symptoms in depression than PHQ-9. This indicates that the Arabic DSSS may capture unique aspects of depression that are not fully captured by the PHQ-9, specifically related to somatic and pain symptoms.

The findings of women scoring higher than men on all subdomains of the A-DSSS could potentially indicate that the A-DSSS scale is more sensitive to capturing gender-specific experiences of depression. The A-DSSS scale may be able to capture somatic symptoms and pain-related dimensions of depression that are more commonly reported by women^35^, and may not be fully captured by the PHQ-9, which primarily focuses on cognitive and emotional symptoms of depression. The gender differences observed in the A-DSSS scores could be influenced by a variety of factors. Biologically, hormonal fluctuations in women, such as those during menstrual cycles, pregnancy, menopause and postpartum period, have been suggested to play a role in the higher prevalence of depression in women^35^. Additionally, sociocultural factors, such as gender roles, societal expectations, and discrimination, may contribute to the higher rates of depression in women^36^. Furthermore, the higher levels of pain and disability reported by women in the sample may be related to the complex relationship between mental health and pain. Depression has been shown to exacerbate pain perception and increase the risk of developing chronic pain conditions^37^. Conversely, chronic pain can also lead to depression and other mental health issues^38^. The higher prevalence of both depression and pain in women could be interrelated, and the A-DSSS scale, by capturing somatic symptoms and pain-related dimensions, may be more sensitive to detecting these interrelated factors in women compared to the PHQ-9^39^. Clinicians and researchers should consider the potential gender differences in the presentation of depression and utilize appropriate assessment tools that capture the unique experiences of individuals, including considering the use of gender-sensitive measures like the A-DSSS scale.

Our findings also revealed that participants who engaged in regular exercise scored significantly lower on the A-DSSS in all subdomains. In fact, there was a consistent negative correlation observed between the MET-min, a measure of exercise intensity, and the A-DSSS score, indicating that higher levels of exercise were associated with lower levels of depressive symptoms across all subdomains of the scale. These findings are consistent with existing literature on the positive effects of exercise on mental health. Previous research has shown that exercise is associated with reduced depressive symptoms and has even been proposed as an adjunctive therapeutic modality for the treatment of depression^40,41^. Exercise has also been shown to be effective in alleviating pain^42^ and improving physical health^43^. Interestingly, this study is the first in the region to investigate the effects of varying exercise intensities, as measured by MET-min, on mental health, and the results are consistent with similar studies conducted in Turkey that utilized similar methods^44,45^. The evidence suggests that exercise may promote neuroplasticity through pathways related to Brain-Derived Neurotrophic Factor (BDNF), a protein that is involved in the growth and maintenance of neurons in the brain^46^. This may explain the beneficial effects of exercise on mental health, including its potential to improve symptoms across all subdomains of depression as assessed by the A-DSSS scale.

The findings of this study highlight the potential advantages of using the A-DSSS as a tool for tracking symptom improvement in all subdomains of depression following the initiation of exercise as a therapeutic modality. However, further research is needed to better understand the underlying mechanisms through which exercise influences mental health and to establish the generalizability of these findings in other populations and settings. Nonetheless, the results suggest that exercise may be a promising adjunctive therapeutic approach for individuals with depression in the Arabic-speaking population.

The results of the study revealed that smokers scored higher on the pain subscale of the A-DSSS compared to non-smokers. This finding is consistent with existing evidence suggesting a bidirectional association between pain and smoking, particularly chronic pain^47^. However, the relationship between depression and smoking is still mixed^48^ and requires further investigation. Assessment of pain is crucial in the evaluation of depression and its somatic symptoms, as pain can often be a prominent feature of depressive disorders^3^. The higher pain scores observed in smokers on the A-DSSS may indicate that smoking could potentially exacerbate pain symptoms in individuals with depression, a hypothesis that is worth testing in the near future.

Our study is the first in the Arabic region to validate the DSSS and explore its correlates. However, our study is limited by the specificity of the sample, primarily consisting of young adults who are university students and single. Consequently, caution should be exercised in generalizing our findings to broader demographics within the Arabic-speaking community. Moreover, the data collection method used in our study, which involved an online self-administered survey, is subject to potential biases, such as selection and response bias that may affect the validity of the results^49^. Furthermore, the application of convenience sampling, a form of non-probability sampling, introduces the potential for sampling bias, thereby compromising the generalizability of our study's findings^50^. To address this limitation, we are actively planning subsequent research with a more diverse and representative sample. The evidence for the psychometric properties of the A-DSSS in this study is contingent on the specified population. While our findings offer preliminary insights, a more comprehensive validation is essential. Future studies will involve a larger and more diverse participant pool, including follow-up data, to establish the scale's reliability and validity across a broader spectrum of individuals within the Arabic-speaking population. Examining the psychometric properties of the DSSS in clinical populations would provide valuable information on its applicability and usefulness in clinical practice. Additionally, conducting longitudinal studies in future research endeavors is crucial. This approach will enable a more in-depth exploration of the temporal dynamics and trajectories of somatic symptoms of depression, providing a comprehensive understanding of their evolution over time within the Arabic-speaking population.

## Conclusion

In summary, the A-DSSS is a valid tool that can enhance the detection and assessment of depression, including associated somatic and pain symptoms. Our findings indicate that men and individuals who engage in more metabolically intense exercises tend to score lower on all A-DSSS subdomains. The A-DSSS is a valuable tool that can be advantageous in cultures where somatic symptoms are commonly expressed in depression, such as in some Asian countries^51–53^ and potentially in Lebanon. These findings contribute to the existing literature on somatic symptoms of depression and highlight the need for a multi-layered approach in the assessment and treatment of individuals with depression, considering both psychological and somatic manifestations. Further research with larger and more diverse samples, including clinical settings, is needed to validate and extend our findings and better understand the complex relationship between depression and somatic symptoms in different cultural contexts.

## Data Availability

Data available upon request from the corresponding author: l.abouabbas@ul.edu.lb.

## Abbreviations

DSSS: Depression and somatic symptoms scale
A-DSSS: Arabic-translated version of the DSSS
PHQ9: Patient health questionnaire-9
HAMD: Hamilton depression rating scale
SS: Somatic subscale
MDD: Major depressive disorder
CVI: Content validity for each item
AUDIT-C: The alcohol use disorder identification test
MET-minute score: Metabolic equivalent minute score
EFA: Exploratory factor analysis
KMO: Kaiser–Meyer–Olkin
CFA: Confirmatory factor analysis
RMSEA: Root mean square error of approximation
CFI: Comparative fit index
CR: Composite reliability
BDNF: Brain-derived neurotrophic factor
